# Magnetic-Dielectric Cantilevers for Atomic Force Microscopy

**DOI:** 10.3390/nano14100874

**Published:** 2024-05-17

**Authors:** Gala Sanchez-Seguame, Hugo Avalos-Sanchez, Jesus Eduardo Lugo, Eduardo Antonio Murillo-Bracamontes, Martha Alicia Palomino-Ovando, Orlando Hernández-Cristobal, José Juan Gervacio-Arciniega, Miller Toledo-Solano

**Affiliations:** 1 Facultad de Ciencias Físico-Matemáticas, Benemérita Universidad Autónoma de Puebla, Av. San Claudio y Av. 18 sur, Col. San Manuel Ciudad Universitaria, Puebla Pue 72570, Mexico; gala.sanchez@alumno.buap.mx (G.S.-S.); hugo.avaloss@alumno.buap.mx (H.A.-S.); eduardo.lugo@sagesentinel.com (J.E.L.); marthap@fcfm.buap.mx (M.A.P.-O.); 2 Faubert Lab, School of Optometry, University of Montreal, Montreal, QC H3T1P1, Canada; 3 Sage-Sentinel Smart Solutions, 1919-1 Tancha, Onna-son, Kunigami-gun, Okinawa 904-0495, Japan; 4 Centro de Nanociencias y Nanotecnología, Universidad Nacional Autónoma de México, Ensenada 22800, Mexico; emurillo@ens.cnyn.unam.mx; 5 Escuela Nacional de Estudios Superiores, Universidad Nacional Autónoma de México, Antigua Carretera a Pátzcuaro 8701, Colonia San José de la Huerta, Morelia 58089, Mexico; ohernandez@enesmorelia.unam.mx; 6 Consejo Nacional de Humanidades, Ciencias y Tecnologías-Facultad de Ciencias Físico-Matemáticas, Benemérita Universidad Autónoma de Puebla, Av. San Claudio y Av. 18 sur, Col. San Manuel Ciudad Universitaria, Puebla Pue 72570, Mexico

**Keywords:** opal–magnetite composite, cantilevers, AFM

## Abstract

Atomic force microscopy (AFM) is a technique that relies on detecting forces at the nanonewton scale. It involves using a cantilever with a tiny tip at one end. This tip interacts with the short- and long-range forces of material surfaces. These cantilevers are typically manufactured with Si or Si_3_N_4_ and synthesized using a lithography technique, which implies a high cost. On the other hand, through simple chemical methods, it is possible to synthesize a magneto-dielectric composite made up of artificial SiO_2_ opals infiltrated with superparamagnetic nanoparticles of Fe_3_O_4_. From these materials, it is possible to obtain tipless cantilevers that can be used in AFM analysis. Tipless cantilevers are an alternative tool in nanoscale exploration, offering a versatile approach to surface analysis. Unlike traditional AFM probes, tipless versions eliminate the challenges associated with tip wear, ensuring prolonged stability during measurements. This makes tipless AFM particularly valuable for imaging delicate or soft samples, as it prevents sample damage and provides precise measurements of topography and mechanical and electromechanical properties. This study presents the results of the characterization of known surfaces using magneto-dielectric cantilevers and commercial cantilevers based on Si. The characterization will be carried out through contact and non-contact topography measurements.

## 1. Introduction

Atomic force microscopy (AFM) is a technique facilitating the exploration of materials’ physical, chemical, electrical, and mechanical attributes at both the microscale and the nanoscale [1]. As a versatile tool for characterization, AFM has been applied in various environments, including room temperature, liquid, and vacuum conditions. The heightened sensitivity of AFM is derived from its capability to discern forces at the pico- and nanonewton levels [2]. The primary sensor characterizing these minute forces in AFM is the cantilever—an anchored beam at one of its extremities. The free end of the cantilever features a sharp tip terminated by a few atoms. Given the diverse applications of AFM microscopies, multiple cantilever designs exist, with rectangular and triangular configurations being the most prevalent. Cantilevers are typically crafted from silicon (Si) and can be further functionalized through coatings to measure specific properties. For instance, in electrical measurements, a conductive coating like platinum (Pt) is applied to the cantilever [3,4]. Gold-coated cantilevers are employed for biological samples [5,6], while a cobalt (Co) coating is requisite for magnetic characterizations [7].

Conversely, tipless AFM cantilevers are frequently employed in specialized applications, such as attaching spheres or other objects to facilitate force spectroscopy measurements. In their study, V. Sboros et al. [8] utilized AFM tipless cantilevers to investigate the mechanical properties and adhesion mechanisms by micro-compressing microbubbles. Furthermore, H. Schillers et al. [9] developed a standardized method for measuring soft and biological samples using colloidal probes comprising spherical SiO_2_ beads with a diameter of 6.62 µm attached to a tipless cantilever. Using the Hertz model for colloidal probes, they extracted the elastic modulus from force indentation data. Additionally, Francesco Tantussi et al. [10] demonstrated the feasibility of positioning and scanning microspheres near the surface using a tipless AFM cantilever. The preeminent fabrication process for microcantilevers involves a top-down lithography approach, incorporating etching steps to remove material [11,12] selectively. Executed in a clean room, this process ensures batch production. Different methods of tipless cantilever synthesis, such as dry film photoresist lithography, bottom-up fabrication using photopolymerizable hydrogel, and focused ion beam lithography, have been previously reported [13,14,15,16,17]. However, the techniques for cantilever fabrication in AFM often elude the grasp of many researchers employing this microscopy method. Due to the persistent wear or contamination endured by cantilevers, replacement becomes imperative.

In this perspective, this study aims to introduce a novel and accessible method for AFM tipless cantilever fabrication. A colloidal crystal based on SiO_2_ artificial opal crystals infiltrated with Fe_3_O_4_ superparamagnetic nanoparticles (NPs) is initially synthesized. This SiO_2_-Fe_3_O_4_ composite with the characteristics of magnetic photonics crystals (MPCs), because of the substantial enhancement of the polar Kerr effect and modification of the Faraday effect [18,19], was obtained using the co-assembly method [20]. These MPCs have potential applications as wide-band ideal optical diodes [21], for enhanced light–matter interaction [22], and recently as a photocatalyst for the degradation of methylene blue [23]. As the composite was prepared on a glass substrate, after the thermal treatment, cantilevers were generated and subsequently coated with either silver (Ag) or aluminum (Al). Mechanically separated from the glass substrate, these cantilevers can be affixed to the AFM silicon chip. The end product is an opal–magnetite cantilever suitable for assessing the surface morphology of materials through AFM. Finally, AFM topography images obtained with commercial and fabricated cantilevers are compared.

## 2. Experimental Details and Results

### 2.1. Synthesis of SiO_2_ Microspheres

SiO_2_ microspheres were synthesized using the Stöber method [24], as described by Santamaria et al. [25]. A 100 mL solution was initially prepared by mixing 1.45 M of 28% ammonium hydroxide (NH_4_OH, J.T. Baker) with 3.6 M of deionized water. Subsequently, another solution of 50 mL was formulated by combining 2.66 M of tetraethyl orthosilicate (TEOS, 98%, Aldrich) with 2.6 M of ethanol (J.T. Baker, 99.9%). These two solutions were stirred separately for 10 min and combined after 2 h of stirring. The resulting SiO_2_ spheres were isolated via centrifugation and subjected to three washes with deionized water. The chemical reaction for sphere synthesis is as follows:

Si(OCH_2_CH_3_)_4_ + 2H_2_O → SiO_2_ + 4CH_3_CH_2_OH

### 2.2. Synthesis of Fe_3_O_4_ Nanoparticles

Taking FeCl_3_ and FeCl_2_ as precursors, the magnetite particle NPs (Fe_3_O_4_) were synthesized by a coprecipitation method from their aqueous solutions at a strongly basic pH (pH = 12) [26]. Initially, the molar ratio maintained between the precursors was Fe^2+^:Fe^3+^ = 1:2 ([Fe^2+^] = 0.25 M and [Fe^3+^] = 0.5 M). Subsequently, at 30 °C, a 2 M NaOH solution was added dropwise to maintain the pH under vigorous stirring in the presence of N^2^ gas. Complete chemical precipitation was achieved after stirring for five hours at 70 °C. Finally, the product was collected after cooling, magnetically separating, and washing thoroughly with deionized water, followed by acetone. The obtained blackish NPs were dried in an oven at 80 °C. The chemical reaction can be expressed as follows:

FeCl_2_ + 2FeCl_3_ + 8NaOH → Fe_3_O_4_ + 8NaCl + 4H_2_O

### 2.3. Synthesis of the SiO_2_-Fe_3_O_4_ for Cantilever 

**Figure 1:**
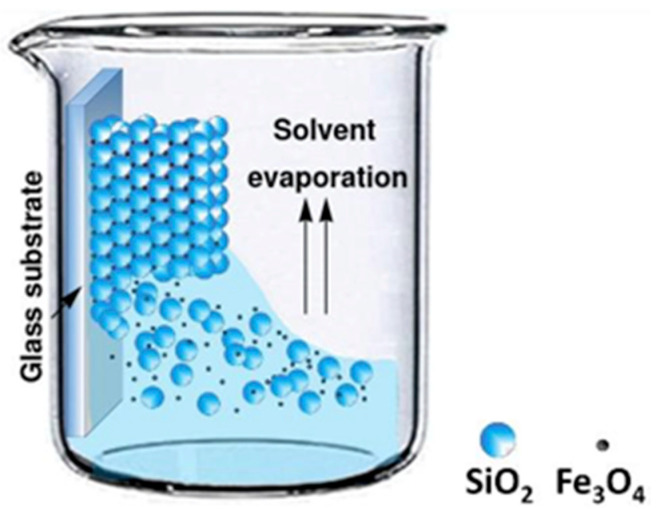
Schematic illustration of the fabrication process of SiO_2_-Fe_3_O_4_-based cantilevers.

Figure 1 shows a schematic illustration of the fabrication process of the SiO_2_-Fe_3_O_4_ composite. In a 50 mL beaker, a solution consisting of 30 mL of 0.066 M colloidal spheres of SiO_2_ and 0.058 M of Fe_3_O_4_ was prepared. Then, a glass substrate of approximately 10 × 25 × 1.5 mm³ was vertically inserted to form a film by evaporating the solvent at 80 °C for 18 h in a muffle (Teralab MA12D). According to the methodology reported by Carmona-Carmona et al. [27], following the evaporation of the water through the voids, the SiO_2_ colloids were packed in an ordered structure under the induction of capillary force. At the same time, Fe_3_O_4_ NPs of a small size compared with the colloidal spheres can easily move in to fill the voids of the colloidal crystal. 

**Figure 2:**
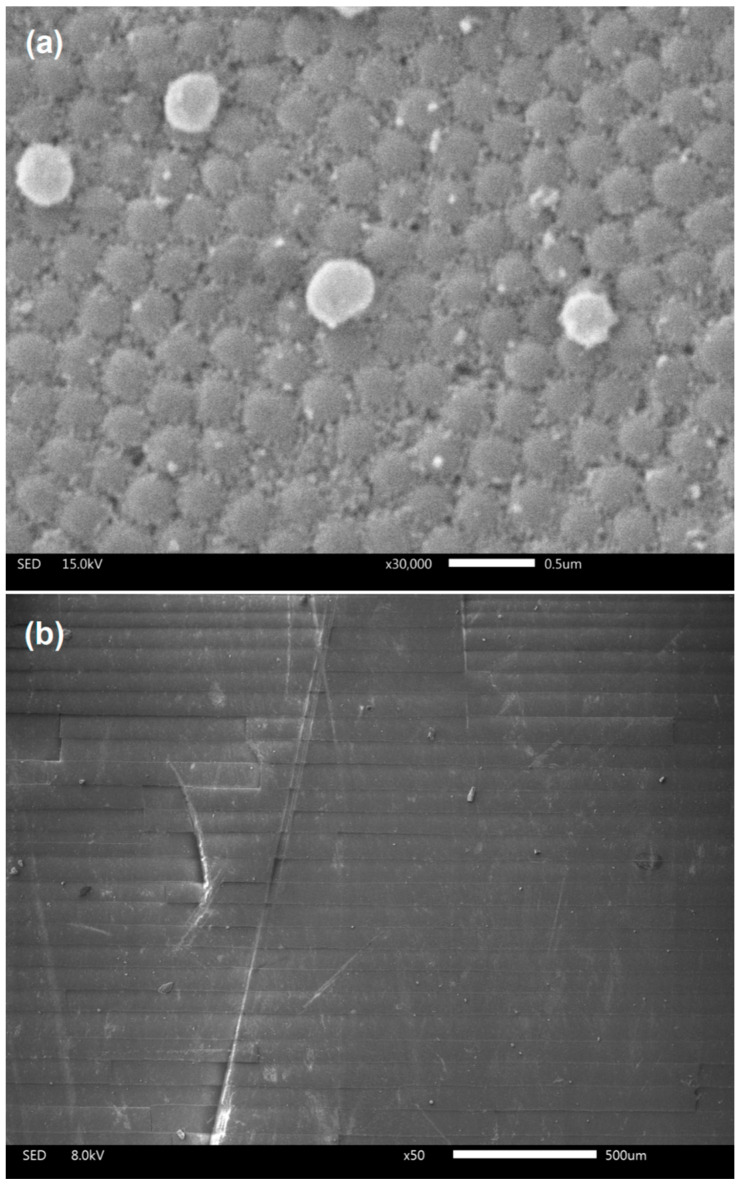
(**a**) SEM micrographs of an opal matrix made from 277 nm diameter SiO_2_ spheres after infiltration with Fe_3_O_4_ NPs. (**b**) SEM micrograph of the SiO_2_-Fe_3_O_4_ cantilevers obtained after the synthesis.

Figure 2a shows the scanning electron microscopy (SEM) image of the internal family surface of the SiO_2_ opal (the average size of a sphere is about 277 ± 10 nm) with the Fe_3_O_4_ NPs well distributed in the voids of the opal. These NPs have a quasi-spherical morphology and an average size of 20 ± 4 nm and exhibit superparamagnetic behavior [27].

Following the synthesis process of the SiO_2_-Fe_3_O_4_ composite, upon the 18 h drying period at 80 °C, fractures become apparent in the structures deposited on the substrate, as illustrated in the SEM image in Figure 2b. The lattice’s shrinkage forms linear cracks while drying the wet-ordered structure. Notably, these cracks frequently align over short distances with crystallographic directions [28].

The dimensions of the cantilevers (Figure 2b) correspond to those of the commercial cantilevers utilized in atomic force microscopy, and their suitability for this application will be demonstrated subsequently. Although the SiO_2_ opal cantilevers were fragile and could break easily, the interaction between SiO_2_ and Fe_3_O_4_ in the SiO_2_-Fe_3_O_4_ cantilevers resulted in better mechanical properties [27]. This enhancement made them suitable for use as cantilevers in atomic force microscopy by making them easier to handle.

### 2.4. Mounting the Cantilever to the AFM Silicon Chip

**Figure 3:**
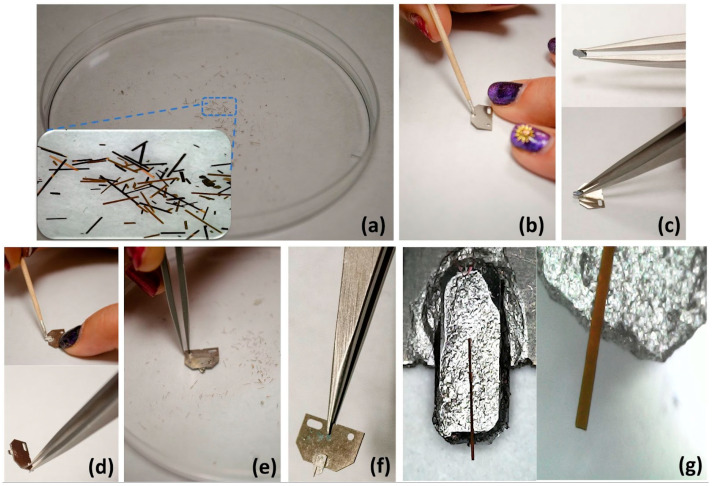
The SiO_2_-Fe_3_O_4_ cantilever is mounted on a silicon chip to obtain a cantilever for AFM. (**a**) Close-up of the scratched-off cantilever on a petri dish. (**b**) Applying silver conducting paint as an adhesive to a silicon chip. (**c**) Attaching a silicon plaque to the silicon chip. (**d**) Applying silver paint to the silicon plaque. (**e**) Picking up a chosen SiO_2_-Fe_3_O_4_ cantilever with the paint-covered plaque. (**f**) Final mounted cantilever arrangement. (**g**) Close-ups of the mounted cantilever were observed with an optic microscope.

The SiO_2_-Fe_3_O_4_ composite is coated with 100 nm of Al by thermal evaporation; this particular procedure is essential in facilitating the reflection of the laser used in the atomic force microscopy (AFM) system. It is worth noting that the thickness of the aluminum coating is considered negligible when compared to the overall thickness of the SiO_2_-Fe_3_O_4_ composite. The SiO_2_-Fe_3_O_4_ film is then scratched with forceps, causing some SiO_2_-Fe_3_O_4_ cantilevers to come off and be deposited into a container, as observed in Figure 3a. The procedure for mounting the SiO_2_-Fe_3_O_4_ cantilevers onto the silicon chips is described below. First, a silicon plaque is affixed to the silicon chip using silver conductive paint as the adhesive (see Figure 3b,c). Subsequently, an appropriate SiO_2_-Fe_3_O_4_ cantilever is selected using an optical microscope and adhered to the plaque, again using silver paint as the adherent (see Figure 3d). The cantilever is picked up from the plate by carefully pressing the paint-covered silicon plaque into the SiO_2_-Fe_3_O_4_, becoming attached to it (see Figure 3e). Forceps are used to handle the materials, and the paint is applied with a wooden toothpick. Finally, the SiO_2_-Fe_3_O_4_ cantilever is ready for use in the AFM system. Details of the final opal cantilever mounted on the silicon chip can be observed in Figure 3g.

### 2.5. SiO_2_-Fe_3_O_4_ Cantilever Calibration

**Figure 4:**
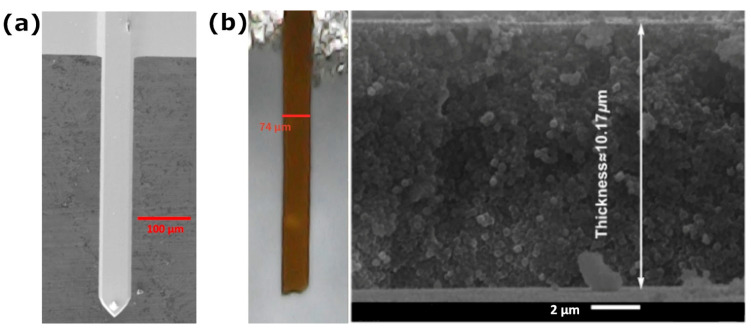
SEM images of (**a**) the commercial cantilever ContactG from Budget Sensors and (**b**) the width and thickness of the SiO_2_-Fe_3_O_4_ cantilever.

The essential characteristics of the cantilevers are their dimensions and the spring constant. The SiO_2_-Fe_3_O_4_ cantilever shown in Figure 3g has a width of approximately 74 μm, a length of 629 μm, and a thickness of 10.17 μm. The spring constant can be obtained using the equation of the point-mass model:

*K = Eab*^3^*/4L*^3^, (1)

where *E* is Young’s modulus, a is the width, *b* the thickness, and *L* is the length. By using the dimensions of the SiO_2_-Fe_3_O_4_ cantilever from Figure 4b with *E* ≈ 58 GPa, the spring constant is then *k* = 4.6 N/m.

Another way to calculate the cantilever spring constant is by using the Sader method [29]. With this method, the spring constant of the opal cantilever is *k*_Sader_ = 1.23 N/m.

Table 1 compares the dimensions and spring constants of the SiO_2_-Fe_3_O_4_ and commercial cantilevers. Here, *w*_0_ is the first eigenmode frequency, and Q is their quality factor.

### 2.6. Atomic Force Microscopy of the Calibration Sample Measured by the Opal Cantilevers

Although the SiO_2_-Fe_3_O_4_ cantilevers are tipless, we have verified that they can be used to characterize, at the very least, the two-dimensional surface of samples with differences in morphology on the order of microns. This is particularly useful, for example, in characterizing biological samples.

#### 2.6.1. Contact Mode

The contact mode in AFM is a microscopy technique where the tip is in contact with the surface, applying a constant force. If higher contact forces are applied, contact with the surface can cause wear to the cantilever. To test the opal cantilevers, we measured the calibration Si grid HS-100MG (111 nm) standard sample using both the SiO_2_-Fe_3_O_4_ and commercial cantilever ContactG from Budget Sensors. This grid features cubes of 6 μm × 6 μm and a height of 111 nm.

**Figure 5:**
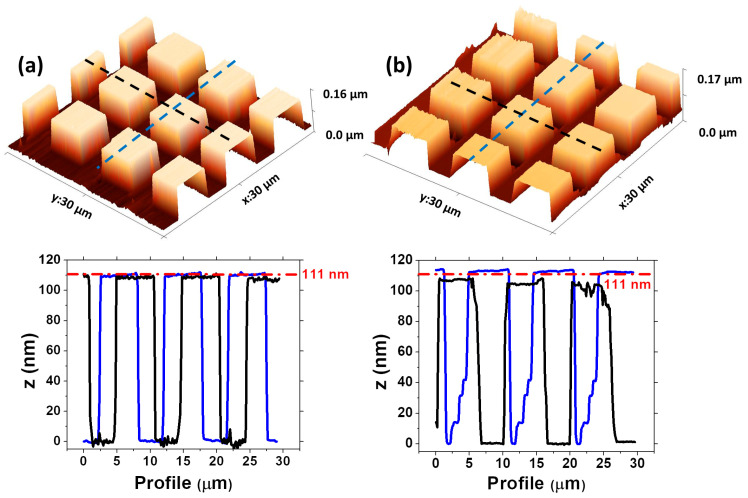
The HS-100MG (111 nm) standard sample measured with (**a**) commercial cantilever ContactG from Budget Sensors and (**b**) SiO_2_-Fe_3_O_4_ cantilever.

In Figure 5a, the calibration grid measured by the commercial tip is shown, with profiles obtained parallel to the x-axis in blue demonstrating better correspondence with the 111 nm height for the standard specification. However, the black profile (taken from the 3D image, Figure 5a) parallel to the y-axis indicates heights for the cubes slightly smaller than 111 nm (red line in the profile graph), suggesting a slight deviation in the commercial cantilever’s performance.

The 3D topography of the calibration grid obtained using the SiO_2_-Fe_3_O_4_ cantilever is shown in Figure 5b, where the cubes are visible. The profile parallel to the x-axis (blue line, Figure 5b) indicates the heights of the cubes slightly taller than 111 nm. In the deeper regions, some artifacts associated with the tipless and multiple contacts can be observed [30]. The profile parallel to the y-axis (black line, Figure 5b) shows heights below 111 nm.

#### 2.6.2. Non-Contact Atomic Force Microscopy

**Figure 6:**
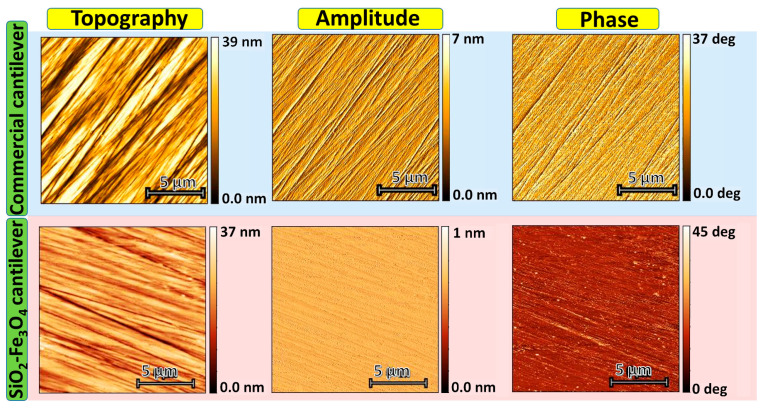
The hard disk sample was measured with the commercial cantilever Tap150Al-G from Budget Sensors and the SiO_2_-Fe_3_O_4_ cantilever.

In the non-contact AFM (NC-AFM) of the Park System AFM, the tip oscillates near the surface in the attractive force regime. Since variations in sample topography result in changes in the tip-sample distance and interaction forces, the amplitude change can be utilized to detect sample topography. Thus, the oscillation amplitude measured at the operating frequency serves as the feedback signal in this mode. The topography in NC-AFM mode was measured on a hard disk sample using both the commercial Tap150Al-G from Budget Sensors and SiO_2_-Fe_3_O_4_ cantilevers. In Figure 6, the topography obtained using a commercial cantilever shows typical details of the hard disk surface and the amplitude and phase show signals associated with the topography. When using the SiO_2_-Fe_3_O_4_ cantilever, the resolution is slightly reduced compared to the topography measured by the commercial tip due to its tipless nature. Moreover, the surface roughness is 5.184 nm and 4.678 nm for the commercial Tap150Al-G and SiO_2_-Fe_3_O_4_ cantilevers, respectively. Some contrast in the amplitude and phase can be detected with the SiO_2_-Fe_3_O_4_ cantilever.

#### 2.6.3. Atomic Force Microscopy of Red Blood Samples

**Figure 7:**
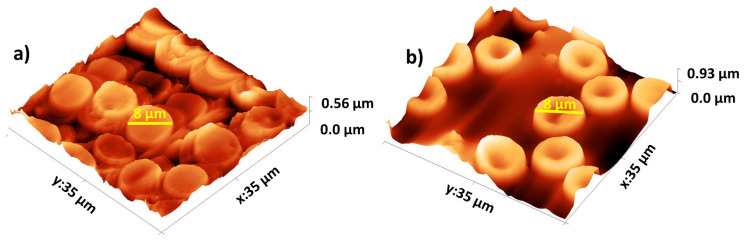
Red blood cells were measured with (**a**) a tipless SiO_2_-Fe_3_O_4_ cantilever and (**b**) a commercial cantilever. The red blood cells’ size and thickness appear consistent in both images. Nonetheless, there is a noticeable disparity in the depth of the center when comparing the commercial cantilever to the red blood cells acquired by the SiO_2_-Fe_3_O_4_ cantilever. The larger contact area of our tipless cantilever is responsible for this difference.

Additionally, the tipless SiO_2_-Fe_3_O_4_ cantilever was utilized to measure a red blood sample, confirming its utility in characterizing the morphology of biological samples. Blood drops were deposited on a glass slide and smeared using another glass slide to prepare the sample. Immediately afterward, the sample’s surface was measured in contact mode AFM using the tipless SiO_2_-Fe_3_O_4_ cantilever. The 3D topography obtained using the tipless SiO_2_-Fe_3_O_4_ cantilever in contact mode of the red blood cells can be observed in Figure 7a. The mean diameter size of the red blood cells was 8 μm, with a thickness of approximately 2 μm, consistent with sizes reported in the literature [31].

Furthermore, a topography image of the red blood cells was taken using a commercial cantilever, as observed in Figure 7b. The red blood cells’ horizontal size and thickness are similar when comparing Figure 7a,b. However, a deeper center is observed using the commercial cantilever in Figure 7b compared to the red blood cells obtained by the SiO_2_-Fe_3_O_4_ cantilever. This difference is due to our tipless cantilever’s larger contact area.

### 2.7. SiO_2_-Fe_3_O_4_ Cantilever Performance

**Figure 8:**
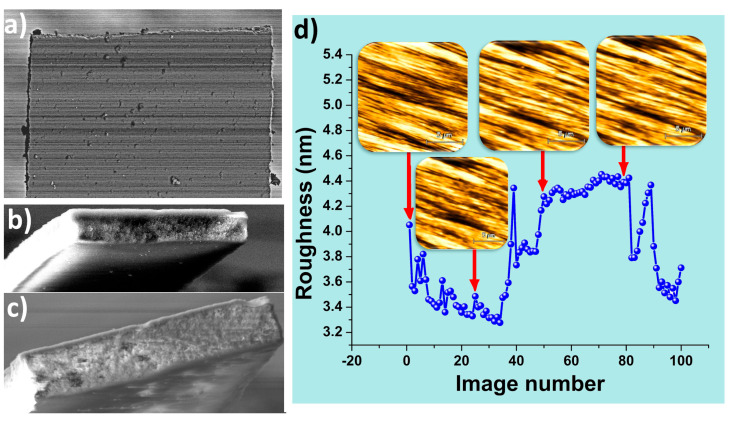
SEM image of (**a**,**b**) the unused SiO_2_-Fe_3_O_4_ cantilever, (**c**) the SiO_2_-Fe_3_O_4_ cantilever until scanning 100 AFM images in contact mode, and (**d**) the roughness of the hard disk surface by using the SiO_2_-Fe_3_O_4_ cantilever, with inset AFM topography of the hard disk.

So far, the proposed SiO_2_-Fe_3_O_4_ tipless cantilevers have demonstrated promising results for atomic force microscopy applications. However, their performance in numerous contact scans still needs to be evaluated. To address this, we initially examined the end of an unused SiO_2_-Fe_3_O_4_ cantilever, as depicted in Figure 8a,b. While some imperfections are visible, there’s no apparent indication of a tip responsible for resolution enhancement, as observed in prior AFM image sections. The contact is probably established between one corner of the SiO_2_-Fe_3_O_4_ cantilever and the sample surface, facilitating AFM images comparable to those obtained with commercial cantilevers. This likelihood stems from mounting the SiO_2_-Fe_3_O_4_ cantilever to the AFM head, ensuring a 16° angle between the SiO_2_-Fe_3_O_4_ cantilever’s length and the horizontal surface. Moreover, due to the manual gluing process, the SiO_2_-Fe_3_O_4_ cantilever may not align perfectly with the silicon chip’s horizontal surface.

On the other hand, the SiO_2_-Fe_3_O_4_ cantilever used does not exhibit significant changes or wear after capturing 100 images in contact mode on the surface of the hard disk, as illustrated in Figure 8c. However, the roughness of the images does display alterations during the 100 contact scans at a resolution of 256 pixels by 256 pixels (see Figure 8d). This outcome suggests that the contact between the SiO_2_-Fe_3_O_4_ cantilever and the sample changes, with the SiO_2_-Fe_3_O_4_ cantilever likely experiencing a minor amount of wear that is nearly indistinguishable when observing the topography images (refer to inset topography insets in Figure 8d).

## 3. Conclusions

The presented study demonstrated the versatile application of tipless SiO_2_-Fe_3_O_4_ cantilevers in atomic force microscopy. The fabrication process, involving the synthesis of SiO_2_ microspheres and deposition of opal films with Fe_3_O_4_ NPs, yielded cantilevers with enhanced mechanical properties suitable for AFM analyses. The utilization of these cantilevers in both contact and non-contact AFM modes was successfully demonstrated for characterizing surfaces, with particular attention to biological samples. The calibration and comparison with commercial cantilevers revealed the potential of the tipless SiO_2_-Fe_3_O_4_ cantilevers to provide meaningful topographical information. The practical application of the SiO_2_-Fe_3_O_4_ cantilever in measuring a red blood sample underscored its effectiveness in characterizing biological specimens. The obtained 3D topography of red blood cells, aligned with literature-reported sizes, attests to the reliability and accuracy of the developed cantilevers.

Additionally, cantilevers of spheres of SiO_2_ were synthesized and tested for AFM measurements, but these SiO_2_ opal cantilevers exhibited poor mechanical properties and were prone to break upon handling. On the other hand, the SiO_2_-Fe_3_O_4_ cantilevers, owing to the interaction between SiO_2_ and Fe_3_O_4_ [27], displayed enhanced mechanical properties. This improvement enabled their manipulation and qualified them for use as cantilevers in atomic force microscopy.

## Figures and Tables

**Table 1:** Specifications of the SiO_2_-Fe_3_O_4_ cantilever compared to the ContactG and Tap150Al-G models from Budget Sensors.

Cantilever	Length (μm)	Width (μm)	Thickness (μm)	*w*_0_ (kHz)	Q	*k* (N/m)	*k*_Sader_ (N/m)
SiO_2_-Fe_3_O_4_	629	74	10.2	21	94	4.6	1.23
ContactG from Budget Sensors	508	57	2.7	15	102	0.2	0.2
Tap150Al-G from Budget Sensors *	125	25	2.1	150	100	4.9	4.9

* Factory values.

## Data Availability

Data are contained within the article.
